# Responsible Innovation in AI-Driven Teledentistry: Ethical, Legal, and Economic Considerations for the Globalization Era

**DOI:** 10.15190/d.2026.3

**Published:** 2026-03-31

**Authors:** Richa Kaushik, Ravindra Rapaka

**Affiliations:** ^1^School of Family Medicine and Public Health Sciences, Wayne State University, Detroit, USA; ^2^University of Connecticut, Storrs, CT, USA

**Keywords:** Teledentistry, Artificial Intelligence (AI), Ethical Considerations, Data Privacy, Healthcare Accessibility, Economic Impact.

## Abstract

Teledentistry has evolved from asynchronous communication to real-time consultations, with adoption accelerating during and post-COVID. Concurrently, AI has been leveraged to enhance diagnostic accuracy, efficiency, and accessibility using machine learning, GANs, and connected devices. This scoping review, conducted using Arksey and O’Malley framework and Joanna Briggs Institute guidance, explores the ethical, legal, and economic considerations of AI-driven teledentistry. Following a PRISMA-ScR compliant screening process by two independent reviewers, 137 studies published between 2018 and 2025 were included. Unlike previous reviews that have primarily focused on clinical applications or the general use of AI in healthcare, this review addresses the ethical, legal, and economic considerations of AI in teledentistry in a single paper. It underscores the importance of explainable AI, explores cross-border regulatory challenges, and discusses possible cost models for adoption in smaller practices. This review indicates that AI-powered teledentistry could enhance diagnostic accuracy, facilitate early detection, improve monitoring, and increase accessibility. Nonetheless, these advantages come with critical concerns including data privacy, potential biases, patient autonomy, accountability, and cost. Addressing these issues through governance, oversight, transparency, and economic viability is essential. AI-enabled teledentistry has the potential to transform dental care delivery, but its integration must be approached with careful consideration of its associated challenges.

## SUMMARY

1. Introduction

1.1 Study Objectives and Novelty

2. Methodology

- 2.1 Eligibility Criteria, Search Strategy

- 2.2 Study Selection and Screening Procedures

- 2.3 PRISMA-ScR Flow Description

- 2.4 Temporal Trends in Publications

- 2.5 Thematic Distribution of Included Studies

- 2.6 Geographic Distribution of Included Studies

3. Ethical Implications in AI-driven Teledentistry

- 3.1 Respecting Patient Autonomy through Informed Consent and Explainable AI in Teledentistry

- 3.2 Beneficence and Non-Maleficence in Safeguarding Data Privacy and Security

- 3.3 Promoting Justice by Mitigating Algorithmic Bias

- 3.4 Illustrative Examples of Algorithmic Bias

- 3.5 Maintaining Professional Integrity in the Era of AI

- 3.6 Evidence of AI Performance in Teledentistry

4. Legal Considerations in AI-Driven Teledentistry

- 4.1 Compliance with Data Protection Laws

- 4.2 Liability in AI-Assisted Clinical Decision-Making

- 4.3 Navigating International Legal Frameworks

- 4.4 Legal Obligations for Informed Consent and Transparency

- 4.5 Cross-Border Legal Challenges

5. Economic Considerations in AI-Driven Teledentistry

- 5.1 Cost-Benefit Analysis of AI Implementation

- 5.2 Economic Challenges and Barriers to Adoption

- 5.3 Reduce Healthcare Costs for Underserved Populations

- 5.4 Financial Incentives to Promote AI Adoption

- 5.5 Innovative Financial Models to Increase Accessibility

6. Discussion

- 6.1 Ethical Considerations

- 6.2 Legal Considerations

- 6.3 Economic Considerations

- 6.4 Risk Assessment, Mitigation, and Cybersecurity in AI-Driven Teledentistry

- 6.5 Real-World Examples of AI Implementation in Dentistry

- 6.6 Strengths and Limitations

- 6.7 Comparison with Other Work

- 6.8 Gaps and Opportunities

7. Future Research Directions and Policy Recommendations

- 7.1 Continuous Monitoring and Improvement of AI Systems

- 7.2 Regular Audits and Ethical Considerations

- 7.3 Multi-Stakeholder Collaboration and Knowledge Sharing

- 7.4 Efficient Software Updates and Implementation

- 7.5 Policy Recommendations

8. Conclusions

## 1. Introduction

Teledentistry is a form of telemedicine, that uses information technology and telecommunications to provide virtual dental care, consultation, education, and public outreach in dentistry^1,2^. It has become an important technique to overcome barriers to oral health care delivery in rural and remote communities where there are provider shortages^2^. Initially, teledentistry was limited to routine appointments and preliminary examinations using asynchronous modalities—where patients supplied data for later review^1^. Technological advancements have since enabled synchronous modalities, allowing dentists and patients to communicate in real time. The COVID-19 pandemic was the main catalyst that has driven this change, as teledentistry became critical to preserve continuity of care in lockdown and reduce SARS-CoV-2 transmission within dental office environments^1,3^.

Building upon these advancements, the integration of artificial intelligence (AI) in teledentistry has made remote dental treatment even more effective and efficient. The use of AI in diagnostic and screening software makes it possible to detect risk factors for oral disease, and help to spot potentially cancerous lesions earlier, particularly in elderly individuals^4,5^. By improving record keeping and triage, AI can help dentists to deal with challenging cases and attend to routine procedures^4^. It also personalized treatments by tailoring individual care plans and offering individual guidance regarding oral health care. By leveraging machine learning, generative adversarial networks (GANs), the Internet of Dental Things (IoT) and smart toothbrushes, AI transforms remote dental care and enables teledentistry systems to be more accessible^5,6^. However, these advancements bring forth important ethical and legal considerations.

Privacy and data security are always at the forefront. All patient information should be encrypted and anonymized under strict regulations such as the General Data Protection Regulation (GDPR) in Europe or Health Insurance Portability and Accountability Act (HIPAA) in US, with data stewardship practices, such as decentralized data sharing, further safeguard patient privacy^7,8^. Algorithmic bias can be misleading or discriminatory, and so AI systems need to be tested against diverse datasets and continually evaluated for validity and consistency^7^. It’s also important to be transparent and explainable, since most AI systems are black-box, which erodes trust and accountability. Both patients and dentists need to know how AI diagnoses or treatment recommendations are calculated^7^. These concerns are being addressed in legislation, such as the European Union’s proposed Artificial Intelligence Act (AIA), which puts an emphasis on trustworthiness and human-centricity in AI applications^9^. Moreover, malpractice laws are also increasing their scope to telemedicine and digital health, as violations of patient privacy and professional negligence can be legally actionable^8^.

Economically, AI-powered teledentistry will initially add to healthcare costs due to the costs of purchasing and installing AI equipment, training expert personnel, and addressing additional issues identified by AI systems^10^. But in the long term, AI could save patients significant money by detecting and preventing dental issues earlier, thereby avoiding expensive procedures and ensuring greater efficacy of dental care. Conducting cost-effectiveness analyses will be critical to understanding these economic costs and providing a guide to apply AI in the clinic^10^.

To understand the technological advancements driving AI-driven teledentistry, **Table 1** provides a comprehensive look at the major AI technologies, their applications and challenges. Machine learning

**Table 1:** AI and Related Technologies in Teledentistry: Functions, Applications, and Challenges. The table summarizes different AI technologies used in teledentistry along with their functions, applications and implementation challenges. It highlights key technologies such as machine learning, CAD/CAM, 3D printing, AI imaging analysis, IoT sensors, augmented reality, natural language processing, predictive analytics, teledentistry platforms, and explainable AI systems

Technology	Function	Application in Teledentistry	Potential Challenges
Machine Learning Algorithms	Process and analyze large datasets to identify patterns and make predictions	Diagnosis of dental conditions, treatment planning, and outcome prediction ^11-13^	Algorithmic bias, need for high-quality training data ^13^
Computer-Aided Design/Computer-Aided Manufacturing (CAD/CAM)	Create digital designs and fabricate dental restorations	Remote design and production of dental prosthetics and restorations ^14^	Integration with existing workflows, technical expertise required ^14^
3D Printing	Produce physical objects from digital designs	Production of dental models, surgical guides, and custom appliances ^14^	Material limitations, quality control ^14^
Artificial Intelligence (AI) Imaging Analysis	Interpret and analyze dental radiographs and images	Early detection of dental diseases, image interpretation ^12,13^	Reliability of AI interpretations, integration with current imaging systems ^11,13^
Internet of Things (IoT) Sensors	Collect real-time data on oral health parameters	Remote monitoring of patient oral health, treatment adjustments ^15^.	Data security, patient privacy concerns ^15,16^
Augmented Reality (AR)	Overlay digital information on the real world	Improved visualization for treatment planning, patient education	Technical limitations, user adoption^14^
Natural Language Processing (NLP)	Analyze and interpret human language	Automated patient communication, voice-controlled dental records	Interpreting dental terminology, multilingual support
Predictive Analytics	Forecast future outcomes based on historical data	Personalized treatment plans, diagnosis for dental diseases	Data quality, ethical considerations^13,14^
Teledentistry Platforms	Enable remote dental consultations and examinations	Virtual dental visits, remote patient monitoring ^14,15^	Network reliability, regulatory compliance ^14,15^
Explainable AI Systems	Provide transparent reasoning for AI decisions	Improving trust in AI-driven diagnoses and treatment recommendations	Difficulty in explaining advanced algorithms, balancing transparency with efficiency ^16^

algorithms, for example, aid in diagnosis and treatment planning, but are subject to algorithmic bias and requirement for quality training data. Augmented reality, in a similar vein, helps treatment visualization but needs to go through technical and user adoption hurdles. Predictive analytics and IoT sensors make treatment options personalized and remote-monitoring possible, but have ethical and privacy issues. This table highlights both the multifaceted nature of AI’s contribution to teledentistry and the obstacles that need to be overcome for seamless adoption.

In summary, although AI-based teledentistry holds great promise for improving access to, the delivery of, and the customization of dental care, it raises ethical, legal, and economic issues as well. Addressing these issues requires collaboration among dentists, artificial intelligence practitioners, ethicists, lawmakers, and economists to ensure that the implementation of AI in teledentistry is conducted responsibly and ethically.

**Figure 1:**
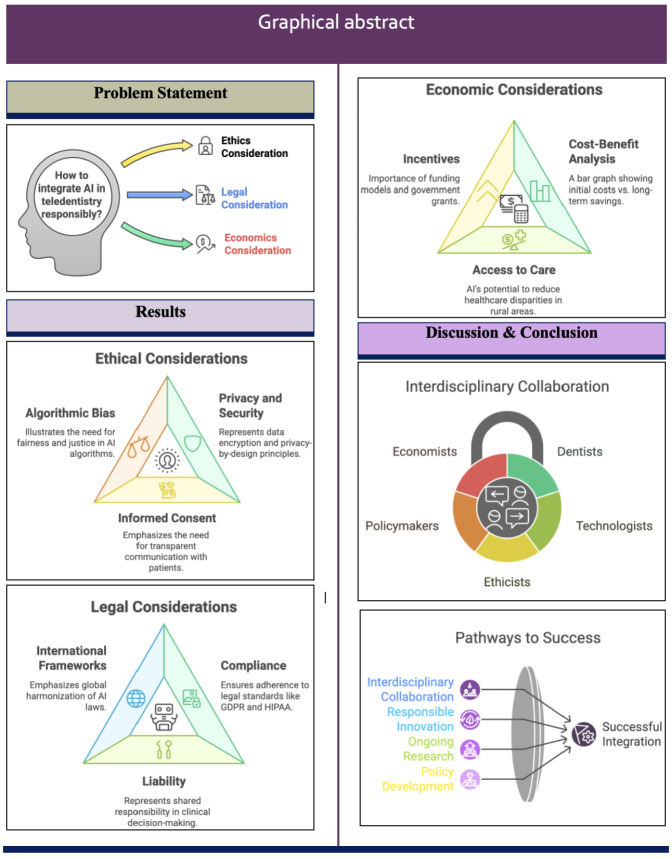
Graphical abstract summarizing the ethical, legal, and economic considerations in AI-driven teledentistry Highlights the problem statement, ethical and legal challenges, economic implications, and pathways to success through interdisciplinary collaboration, policy development, and responsible innovation.

To give a full view of the ethical, legal and economic issues discussed in this article, **Figure 1** represents the essential obstacles and cross-disciplinary routes to an ethical deployment of AI in teledentistry. It is a visual representation of the problem statement, findings and solutions covered in the manuscript.

### 1.1 Study Objectives and Novelty

This scoping review explores the ethical, legal, and economic implications of using artificial intelligence in teledentistry. In particular, it seeks to: investigate the key ethical issues, including patient autonomy, consent, data privacy, bias, and accountability; examine the legal and regulatory considerations, including data security, liability, and cross-border practice; evaluate the economic factors, including cost, accessibility, and affordability in underserved contexts; analyze case studies where AI has been implemented in teledentistry; and identify evidence gaps, challenges, and priorities for future research.

This review builds upon the existing literature by bringing these aspects together in the specific context of teledentistry. Where previous reviews have focused on clinical performance, technical applications, or general AI challenges in medicine or dentistry, this review integrates ethical, legal, and economic considerations with an emphasis on practical implementation issues such as explainability, informed consent, cross-jurisdictional regulations, and affordability for smaller practices.

This study, therefore, aims to better understand the potential and limits of AI teledentistry and to enable more ethical and pragmatic adoption.

## 2. Methodology

This scoping review addresses the ethical, legal, and economic issues related to the use of AI in teledentistry. Arksey and O’Malley’s framework and the Joanna Briggs Institute (JBI) guidelines for scoping reviews were followed. The review protocol was registered on the Open Science Framework (OSF) (https://osf.io/hqa58/).

### 2.1 Eligibility Criteria, Search Strategy

Peer-reviewed articles, conference papers, reviews, case studies, and expert opinions published in English between 2018 and 2025 were eligible for inclusion. Searches were conducted in PubMed, Scopus, arXiv, and IEEE Xplore, as well as Google Scholar. Grey literature was included to ensure coverage of relevant legal and policy documents. Many of the regulations discussed in this review, HIPAA, GDPR, national privacy laws, digital health strategies, and international guidelines, are published directly by governments, statutory agencies, and international bodies rather than in academic journals.

### 2.2 Study Selection and Screening Procedures

All retrieved records were compiled, and duplicate records were removed before the screening process. To ensure a rigorous and transparent approach to the selection process, the following procedures were adopted:

1. **Two-Stage Screening:** Two-stage screening was conducted by carrying out the process of title and abstract screening, followed by a thorough full-text screening process.

2. **Reviewer Roles:** Two reviewers screened the identified records against the predetermined inclusion and exclusion criteria.

3. **Conflict Resolution:** In case of doubt or disagreement over the inclusion of studies, a repeated process of reviewing the full text was conducted to ensure a collaborative approach to resolving conflicts. In case of persistent conflict of interest, the final decision was made by mutual discussion between the two authors.

### 2.3 PRISMA-ScR Flow Description

**Figure 2:**
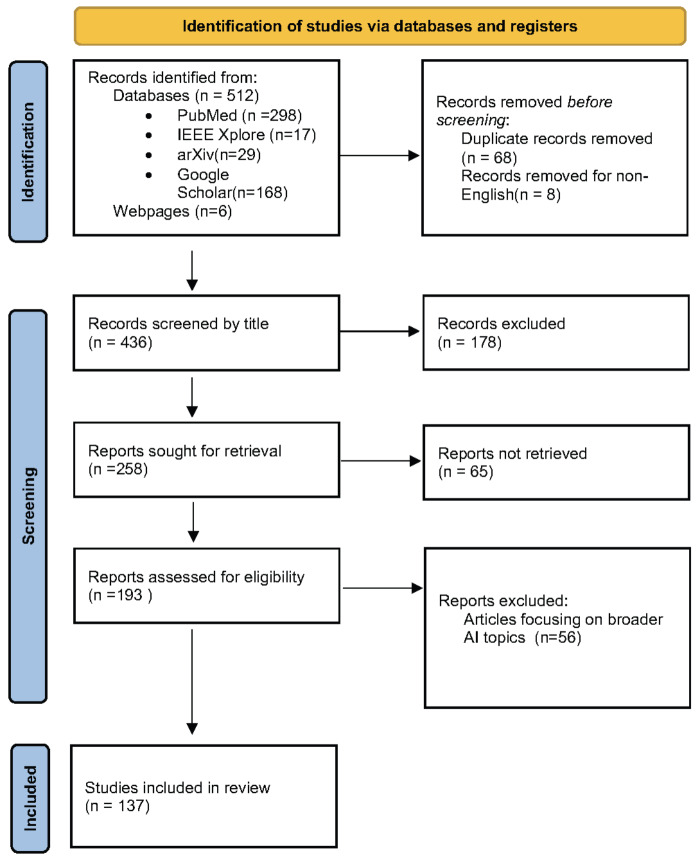
PRISMA Flow Diagram of Study Selection Process. This flow chart explains how the study is identified, screened, assessed for eligibility and included for the review. Records were identified in databases and registers, such as PubMed, IEEE Xplore, arXiv and Google Scholar. There were 137 studies that were taken up for the final review. The methodology follows PRISMA guidelines^138^ (Source: Page MJ, et al., BMJ 2021;372:n71). *Consider, if feasible to do so, reporting the number of records identified from each database or register searched (rather than the total number across all databases/registers). **If automation tools were used, indicate how many records were excluded by a human and how many were excluded by automation tools. Source: Page MJ, et al. BMJ 2021;372:n71. doi: 10.1136/bmj.n71. This work is licensed under CC BY 4.0. To view a copy of this license, visit https://creativecommons.org/licenses/by/4.0/

The study selection results are summarized in the PRISMA-ScR flow diagram (**Figure 2**).

· **Identification:** A total of 512 records were initially identified through database searches.

· **Screening:** After removing 68 duplicates and 8 non-English records, 436 records remained for title and abstract screening. Of these, 178 records were excluded for failing to meet basic relevance criteria.

· **Eligibility:** From the remaining 258 reports sought for retrieval, 193 articles underwent a rigorous full-text assessment for eligibility. During this phase, 56 articles were excluded because they focused on broader AI topics rather than the specific ethical, legal, or economic intersection with teledentistry.

· **Inclusion:** Ultimately, 137 studies met all eligibility criteria and were included in the final qualitative synthesis.

### 2.4 Temporal Trends in Publications

The pattern of published research articles indicates an increasing trend in recent years. The number of published articles was relatively lower until 2021. There were fewer than ten articles published in previous years. A significant increasing trend was observed from 2022 (n = 16), and an increasing pattern was observed in 2023 (n = 28). The highest number of articles was published in 2024 (n = 67), indicating an increasing pattern in research activities in this area. A smaller number of studies were identified in 2025 (n = 9), reflecting the partial coverage of the publication year at the time of data collection (**Table 2** and **Table 3**).

**Table 2:** Distribution of included studies by publication year and country/region This table summarizes the geographic and temporal distribution of the 137 studies included in the review. Studies were grouped by publication year and by country or region of origin. “Global / Multi-country” refers to studies conducted across multiple countries or framed at an international level. “Others” includes countries with smaller individual publication counts that were grouped for presentation. “Before 2022” includes studies published 2018-2022, including one article published before 2018

Year	Global / Multi-country	US	India	Italy	Others	Total
Before 2022	4	3	2	2	6	17
2022	7	1	1	1	6	16
2023	12	5	1	2	8	28
2024	32	10	7	1	17	67
2025	7	1	0	0	1	9
Total	62	20	11	6	38	137

From the pattern, it is evident that research activities in this area have been increasing over time, especially after 2021. The increasing pattern might be due to the overall adoption of digital health technologies and increasing research activities in artificial intelligence in healthcare and dentistry.

### 2.5 Thematic Distribution of Included Studies

Research on AI-powered teledentistry is focused primarily on clinical and technological implementation and on ethical and legal considerations, with fewer addressing economic aspects. Clinical and technological implementation is the largest category (n = 58), reflecting strong interest in AI use for diagnosis, treatment, and workflow-related tasks. General and conceptual papers also constitute a large portion (n = 29) of the articles, mostly addressing AI integration, design, and digital health more broadly. Ethical and legal concerns are also a major focus (n = 46) (**Table 3**).

### 2.6 Geographic Distribution of Included Studies

The studies indicate a clear international orientation in the research on AI and teledentistry. The majority of the studies (n = 62) were carried out in a global/multi-country environment, which points to the importance of digital health innovation in a global environment (**Table 2**).

**Table 3:** Distribution of included studies by thematic category and publication year This table presents the thematic distribution of the 137 included studies across publication years. Studies were classified into four main thematic categories: Clinical and Technological Implementation, Ethical and Legal Considerations, Economic Considerations, and General and Conceptual. “Before 2022” includes studies published 2018-2022, including one article published before 2018.

Theme	<2022	2022	2023	2024	2025	Grand Total
Clinical and Technological Implementation	8	8	6	33	3	58
Economic Considerations	1	0	1	2	0	4
Ethical and Legal Considerations	1	4	14	22	5	46
General and Conceptual	7	4	7	10	1	29
Grand Total	17	16	28	67	9	137

Regarding the specific countries, the US was the leader in the number of studies carried out, with 20 studies, followed by India with 11 studies, and Italy with 6 studies. The remaining countries (n = 38) were lumped together, reflecting the wide geographic distribution of the research on AI in health, as well as the varying degrees of research output in the specific area.

Regarding the concept of responsible innovation , the fact that the majority of the studies were carried out in a multi-country environment points to the increasing cooperation between different countries in the context of the challenges related to the use of AI in health, as well as the need to address the specific challenges in the context of teledentistry in a harmonized way.

## 3. Ethical Implications in AI-driven Teledentistry

Teledentistry, with the rise of artificial intelligence (AI), raises various ethical dilemmas that must be carefully managed to protect guiding principles of patient autonomy, beneficence, non-maleficence and justice. This section examines ethical issues relevant to AI and teledentistry: informed consent, data privacy and security, algorithmic bias mitigation, and professional integrity when it comes to AI-supported clinical decision making.

### 3.1 Respecting Patient Autonomy through Informed Consent and Explainable AI in Teledentistry

In ethical medicine, patient autonomy requires that patients be fully informed and give consent to their care. The use of AI in teledentistry creates new challenges for patient autonomy. The black box nature of AI affects transparency as well as patients' understanding of the diagnosis and hence compromising the informed consent process^17^. The "black box" problem surfaces when advanced AI models, like deep learning neural networks, make internal decision-making processes difficult to understand^17^. In teledentistry, this means a patient gets an AI-generated diagnosis or treatment recommendation without knowing how AI reached its conclusion. This lack of transparency can undermine patient trust and also make it hard to identify and address AI errors or biases^17^. This also makes it harder to meet medical decision-making transparency regulations. As informed consent requires patients to understand the nature, risks, and benefits of the suggested intervention, it poses ethical questions regarding patient autonomy^17^.

Maintaining patient autonomy requires transparent communication that clearly explains AI's contribution to patient care^18^. The patients should be well informed about how AI machines analyze data, diagnose, and recommend treatments. Giving patients the option to consult with human dentists or get a second opinion strengthens their medical choices^19^. Given that AI technologies change over time, informed consent must be considered an ongoing conversation, not a single moment in time.

The emerging field of Explainable AI (XAI) offers approaches to make AI decision-making more transparent and interpretable. Several XAI methods are useful for teledentistry:

· LIME (Local Interpretable Model-agnostic Explanations): LIME builds easy-to-understand local models near specific data points to explain AI predictions^20^. In teledentistry, it highlights image areas that influenced the AI’s diagnosis, showing where it identified the dental condition.

· SHAP (SHapley Additive exPlanations): Using game theory principles, SHAP measures the influence of each input feature over the prediction result^20^. SHAP analysis shows which dental problems (tooth discoloration, enamel erosion, gum recession, bone loss) affect an AI diagnosis most strongly.

· Grad-CAM (Gradient-weighted Class Activation Mapping): Using heatmaps, Grad-CAM creates visual explanations by showing where an AI system focuses its attention during image analysis^20^. In teledentistry, Grad-CAM can be used to highlight which parts of a dental image the AI system analyzes to find caries, lesions and other issues.

· Decision Trees: Decision trees may not deliver accurate results than advanced models but they provide a transparent way to see how decisions are made through their flowchart like representation^21^. AI systems can use these simpler models for basic dental diagnosis or to provide a high-level overview of the factors considered by more complex AI systems.

By using these XAI methods dentists and patients can better understand how AI systems make medical decisions. XAI tools by themselves cannot guarantee patients fully understand the decisions made by an AI system. Some explanations may be too technical for patients, and XAI may not properly reflect the AI model's complexity.

Hence, in AI-driven teledentistry systems, human input and second opinions are equally important. It includes:

· Human-in-the-loop systems generally engage a dentist in decision-making. So, dental professionals can examine, alter, or veto AI recommendations^20^. Clinical expertise and judgment are vital in this approach.

· Confidence Thresholds: AI systems can detect cases that need human evaluation when their prediction certainty drops below set thresholds^20^.

· Patient-Initiated Second Opinions: Teledentistry systems should let patients seek a second opinion through human dentists, this gives patients an opportunity to seek further clarification and reassurance.

· Collaborative Decision-Making: AI should support dentists by providing assistance while preserving traditional dentist-patient interactions. Dentists must learn to present AI results to patients by showing how AI supports patient diagnosis and treatment.

AI teledentistry systems should include continuous learning and feedback mechanisms that let dental professionals and patients provide regular feedback to improve the model's performance by enhancing its algorithms for future use. Patients need to understand how their medical care uses AI technology. Therefore, patients should be educated on these AI tools as well on the role they serve in their healthcare. Informed consent in AI requires ongoing discussion and research.

Teledentistry has the potential to utilize AI ethically by maintaining the principles of consent, autonomy, and transparency through the inclusion of XAI along with strong human oversight, feedback, and patient engagement mechanisms.

### 3.2 Beneficence and Non-Maleficence in Safeguarding Data Privacy and Security

It is ethical principles of beneficence and non-maleficence that require health care professionals to act in the patient’s interest and avoid harm. Teledentistry AI systems need to process massive amounts of data from patients such as the dental records and the imaging which makes data breaches and thefts more likely^22^. These breaches can cause a lot of damage, such as the loss of trust of patients and the theft of information.

The "privacy by design" principle is a must in order to ethically protect patient data^23^. This involves integrating strong data protection such as encryption, secured storage and strict access controls from the inception of AI system development^24^. Regular checks and audits can detect the bugs and comply with the data security policies. Further, informing patients about the use, disclosure, and security of their data increases transparency and patient-provider collaboration^22^.

### 3.3 Promoting Justice by Mitigating Algorithmic Bias

The ethical principle of justice demands equality in health care provision. Artificial intelligence algorithms could also continue or amplify biases because of unrepresentative training data or poor design, and lead to differences in treatment outcomes among patients^25^. Underrepresented groups might, for instance, get poorer diagnoses or inadequate treatments and so contribute to health inequalities.

For the sake of justice, we should use different and representative datasets to train AI so that algorithms perform similarly on different groups^26,27^. Biases can be detected and corrected through implementing robust testing and validation processes^28^. Transparent, explainable AI systems improve judgmental transparency and enable stakeholders to recognize and overcome biases ^27^. The AI systems need to be monitored and continually updated in order to remain fair and keep up with evolving patient populations^29^.

### 3.4 Illustrative Examples of Algorithmic Bias

Algorithmic bias, where AI systems show different results between different demographic groups, creates a major concern in healthcare applications, including teledentistry. While case studies in AI-driven teledentistry are only emerging, research from similar medical fields gives insight on such biases.

An AI system for dermatology failed to maintain consistent accuracy because it processed data from an unknown medical setting^30^. The findings of this study can be extrapolated to teledentistry AI systems that can also show reduced accuracy in diagnostics for patients belonging to underrepresented groups. Adding further to this discussion, in a recent research article, 590,000 chest radiograph readings showed that AI systems perform differently across patient groups when recognizing cardiomegaly, pleural effusion, pneumonia, and atelectasis^31^.

The literature review shows that these performance differences stem from multiple root causes. The primary reason behind performance variations comes from differences in data distribution across clinical settings independent of patient demographics or imaging methods^30^. Another significant contributor to these performance differences is lack of representation of certain demographic groups in training datasets^30^. AI models trained in one ethnic group perform less accurately when used across other populations.

The implications of these findings for teledentistry are significant. AI systems trained on biased datasets produce different results across patient groups. Furthermore, the results of AI systems can vary depending on both the expertise of the person taking the dental images as well as on the tools that were used in teledentistry setups.

The researchers and developers must focus on developing methods to avoid these possible biases. First step requires creating training datasets that represent all demographic groups and oral health disorders^32-34^. This may demand multi-center data collection efforts to obtain data from diverse geographic and demographic contexts^35^. Using Generative Adversarial Networks (GANs) can create synthetic data to improve datasets, particularly for underrepresented groups^33^.

Identifying and addressing bias patterns must become standard practice. The DebiasEdu framework demonstrates how to identify and address biases through gradient-based bias identification mechanisms in educational systems^36^ and similar approaches can help teledentistry reduce bias. The three fairness standards- statistical parity, equal opportunity, and predictive parity also play critical roles in evaluating and mitigating biases^37^. Thus, regular auditing of AI models should include different demographic subgroups and clinical settings to make sure of equitable diagnostic accuracy^38^.

Additionally, regular monitoring and updating of datasets from underrepresented groups is necessary to maintain their representativeness^39^. Transparent reporting of dataset composition, demographic and other descriptive details is also important for enabling external evaluation and improvement of AI systems^34^.

To summarize, though specific instances of algorithmic bias in AI-driven teledentistry are currently limited, data from related domains emphasizes the possibility of such biases. Effective solutions to these problems require a complete strategy that uses diverse datasets, bias mitigation techniques, and ongoing monitoring. By using these strategies teledentistry can ensure that AI systems provide equitable and accurate diagnostic support across all patient populations.

### 3.5 Maintaining Professional Integrity in the Era of AI

Healthcare professionals are morally responsible to use their expertise in appropriate ways and keep patient’s welfare at the forefront. When AI is part of clinical decision-making, there is a risk that it will become too reliant on technology and thus undermine clinician discretion^40^. Practitioners are at risk of submitting too much to AI recommendations without due consideration, and the patient’s care could be negatively affected.

In order to retain professional integrity, it is essential to ensure that AI is used as a complement to rather than an alternative to clinical skills^40,41^. Doctors should continue to actively contribute to decision-making by evaluating AI-driven insights and integrating them with their expertise. By setting clear rules and standards of ethics for AI-based clinical practice, it can be possible to define the profession’s responsibilities and safeguard the quality and safety of care.

**Figure 3:**
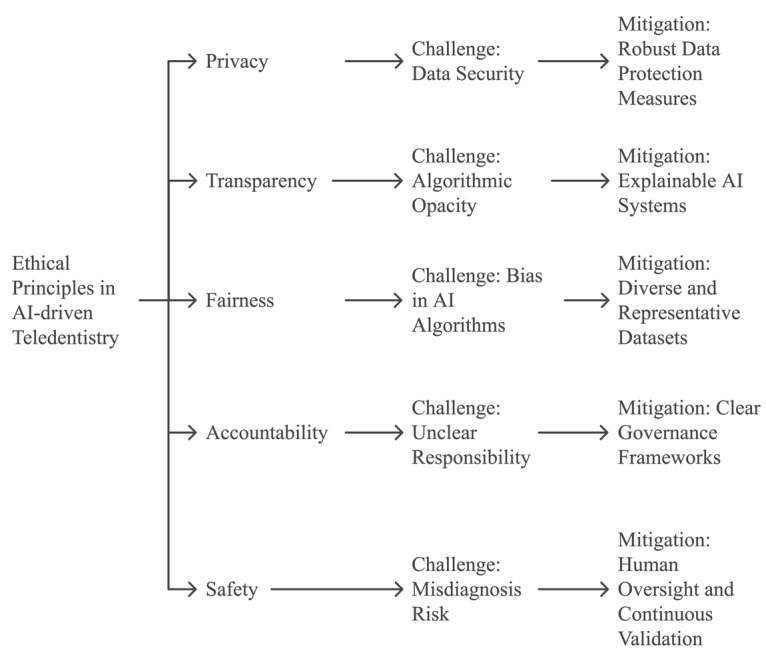
A visual representation of ethical principles and challenges in AI-driven teledentistry An illustration of the ethical issues and challenges with AI-enabled teledentistry. This figure shows important ethical issues like transparency, data privacy and security, algorithmic bias, and accountability and how they’re being posed and tackled. The graphics can help us see how these principles play out under AI-driven teledentistry.

**Figure 3** further explains how ethical principles and challenges of AI-based teledentistry come together. This figure details the key ethical considerations, such as informed consent, data privacy and security, algorithmic bias and professional ethics. It also articulates the challenges and potential mitigations associated with this, in a quick summary of how such principles shape the ethical landscape of AI-enhanced teledentistry.

### 3.6 Evidence of AI Performance in Teledentistry

Several recent studies have quantitatively addressed the use of AI in teledentistry by comparing its diagnostic performance with that of human clinicians. Overall, these studies indicate that AI has the potential to enhance the accuracy and efficiency of dental diagnosis, especially for common conditions such as periapical lesions, dental caries, and periodontal disease. However, performance is task-dependent, and the reported results exhibit considerable variability depending on the imaging modality, the specific diagnosis, the algorithm employed, and the dataset used for training and testing.

AI has demonstrated promising performance in detecting periapical lesions, albeit with varying results depending on the image type. Kazimierczak et al.^42^ tested Diagnocat for identifying periapical lesions on OPGs and CBCT images. On OPGs, the AI achieved 97.01% accuracy and 98.43% specificity, but only 33.33% sensitivity. Human readers achieved higher sensitivity of 66.67%, but lower specificity of 96.82% on OPGs^42^. On CBCT images, the same AI achieved 99.35% accuracy, 77.78% sensitivity and 99.83% specificity^42^. Another investigated AI model achieved 89.6% accuracy, 86.5% sensitivity and 88.1% specificity for periapical periodontitis detection, outperforming one radiologist but not the other with superior diagnostic performance^43^.

AI has also shown promising results for caries detection, albeit with variable performance. In a systematic review, Abesi et al.^44^ reported sensitivities ranging from 97.76% to 99.26%, specificities from 92% to 99.42%, and accuracies from 89.47% to 100%. However, other reported performances are more modest. Using bitewing radiographs, deep learning showed a sensitivity of 0.77 and an AUC of 0.779, versus an AUC of 0.886 for dentists^45^. For smartphone images, YOLOv3 had a sensitivity of 87.4% for cavitated caries but only 36.9% for visually non-cavitated carious lesions, while Faster R-CNN had a sensitivity of 71.4% and 26.0% for cavitated and non-cavitated lesions, respectively^46^. Although specificity was above 86% for cavitated lesions and above 71% for non-cavitated lesions, the large drop in sensitivity suggests these models may be better at ruling in obvious disease rather than detecting early-stage changes. An umbrella review found that AI accuracy for caries diagnosis ranged from 0.733 to 0.986 across datasets^47^. In another publication, an AI system demonstrated 92% sensitivity, 85% specificity, and 88% accuracy, comparable to clinical values of 86%, 90%, and 88%, respectively^48^.

For periodontal disease, the evidence is somewhat more consistent, though still dependent on study design and imaging parameters. Choi et al.^49^ reported pooled sensitivity and specificity of 87% (95% CI: 80% to 92%) and 92% (95% CI: 88% to 95%), respectively, for AI models detecting alveolar bone loss and periodontitis on dental radiographs. Likewise, R-PBL’s performance for detecting periodontitis using panoramic radiographs in accordance with the 2018 case definition was notably consistent^50^. In comparison to the variability observed in caries detection, these figures indicate more stable performance for radiograph-based periodontal assessment, although external validation is required.

Across these diagnostic tasks, a notable trend is the high specificity, yet variable sensitivity exhibited by many AI systems. This trend is exemplified in periapical lesion detection on OPG images, where specificity reached 98.43% but sensitivity was only 33.33%^42^, and in caries detection, where many systems showed dramatically reduced performance for non-cavitated lesions^46^. This suggests that some AI systems might be better suited for confirming obvious pathology rather than identifying the subtle disease. This is likely related to their training data.

The variation between studies also seems to reflect the impact of imaging modality and algorithm choice. CBCT images contain more anatomical detail than OPGs, which may explain the superior performance of AI models on CBCTs for periapical lesion detection^42^. Similarly, smartphone intraoral images may have more variation in illumination, angle, and image quality than radiographs, making consistent detection more difficult, particularly for early lesions^46^. The difference in sensitivity between YOLOv3 and Faster R-CNN also suggests that algorithm architecture can impact sensitivity when lesions are visually ambiguous^46^.

Another key consideration is dataset bias and generalizability. AI models trained and tested within a single clinical setting may perform well locally but not necessarily in other institutions. Black-box models are particularly susceptible to data shifts (e.g. image acquisition, disease prevalence, annotation style), so multi-institutional validation results are critically important. Although examples from other medical imaging tasks (e.g. osteoarthritis and COVID-19 classification) have demonstrated that AI can robustly generalize to large and geographically diverse datasets^51,52^, large-scale external validation is needed before this can be confidently asserted for teledentistry.

Taken as a whole, the available evidence suggests that AI can match or even exceed clinician diagnostic performance for certain teledentistry applications. Yet, the literature also highlights the context-dependency of reported performance. Interpreting AI accuracy therefore demands consideration of not only the summary metrics, but also the sensitivity-specificity trade-off, the image type, the lesion type, the algorithm design, and the diversity of the training and test datasets.

## 4. Legal Considerations in AI-Driven Teledentistry

The use of AI in teledentistry raises a number of complicated legal issues, including obligations regarding informed consent, liability in clinical decision-making aided by AI, differences in international legal frameworks, and compliance with data protection laws. Protecting patient rights, maintaining regulatory compliance, and encouraging responsible innovation in the teledentistry industry all depend on addressing these legal issues.

### 4.1 Compliance with Data Protection Laws

Teledentistry AI systems access private patient data, which requires a stringent compliance to regulations regarding data privacy, including the General Data Protection Regulation (GDPR) and Health Insurance Portability and Accountability Act (HIPAA)^53,54^. These laws mandate high data privacy and security requirements, which require businesses to take appropriate precautions against unauthorized access, data breaches, and misuse of information.

Compliance to the law is not only technical protection but also creating data handling policies and procedures. This includes obtaining patient-consent to the processing of data, data minimization and patient rights for access, correction or deletion of their data^55^. It is imperative to have strong cybersecurity protection of AI systems against adversity and confidentiality of patient data^56^. Data ownership and control rights need to be clearly defined, so that patients understand and are in control of the use and disclosure of their data^57^.

### 4.2 Liability in AI-Assisted Clinical Decision-Making

Legal responsibility is a major concern when it comes to AI-supported clinical decision-making. There are concerns about who should be held accountable if an AI system leads to a incorrect diagnosis or treatment recommendation: the dental practitioner, the AI developer, or the organization using the technology^58,59^.

Legal structures will need to adjust to this, perhaps through a framework of clearly defined norms of care for AI^58^. Dentists might be required to verify AI recommendations and cannot just accept AI outputs without proper validation. The agreements between dentists and AI providers could specify responsibility and indemnification in the event of an AI-system error. The regulators could also need to issue advice on legal obligations and professional duties in the adoption of AI technologies.

### 4.3 Navigating International Legal Frameworks

Navigating disparate legal requirements across jurisdictions is made more difficult by the worldwide scope of AI and teledentistry services. Different countries have different approaches to regulating AI; some have adopted horizontal frameworks that address AI across sectors, while others have implemented healthcare regulations that are specific to a given context^60,61^.

For teledentistry providers working on a global basis, it is important to be familiar with the regulations of the countries where they are providing their services. This could include compliance with a variety of data protection regulations, medical device regulations, and professional licensing. Global partnership and legislative efforts can encourage ethical and legal applications of AI around the world and help remove barriers to transnational teledentistry services^62^.

To give a more comprehensive picture of legal considerations related to AI-based teledentistry, **Table 4**^63-68^compares the regulatory frameworks in the major countries (US, EU, Canada, Australia, India, and global guidelines). Table highlights relevant laws, including HIPAA in the US and GDPR in the EU, including data privacy, patient consent and AI transparency requirements. It also identifies the most common implementation issues, such as adapting regulations to rapid technological advancements, cross-border compliance and jurisdictional variations. This comparative analysis illustrates the critical need for global harmonization and innovation-friendly legal frameworks to support the ethical and effective integration of AI in teledentistry.

**Table 4:** Comparison of Legal Frameworks for Teledentistry Across Different Regions. The table offers a comparative review of teledentistry legal frameworks across the United States, European Union, Canada, Australia, India and includes World Health Organization (WHO) global guidelines. The table identifies essential elements of each regulation and discusses the obstacles in applying these legal standards for AI-driven teledentistry.

Relevant Regulation	Region	Key Provisions	Challenges in Implementation
HIPAA (Health Insurance Portability and Accountability Act)^63^	United States	- Data privacy and security requirements - Patient consent for telehealth services - Secure transmission of health information	- Ensuring AI systems comply with data protection standards - Maintaining patient privacy in remote consultations
GDPR (General Data Protection Regulation)^64^	European Union	- Strict data protection and privacy rules - Right to explanation for AI-driven decisions - Data minimization and purpose limitation	- Balancing AI innovation with stringent data protection - Providing clear explanations for complex AI algorithms
PIPEDA (Personal Information Protection and Electronic Documents Act)^65^	Canada	- Consent for collection and use of personal information - Safeguarding personal health information - Transparency in AI-driven processes	- Adapting regulations to rapidly evolving AI technologies - Ensuring cross-border data compliance
Privacy Act 1988^66^	Australia	- Australian Privacy Principles for health information - Secure storage and transmission of health data - Patient access to their health records	- Addressing jurisdictional issues in teledentistry - Updating regulations to keep pace with AI advancements
Digital Information Security in Healthcare Act (DISHA)^67^	India	- Protection of digital health data - Consent-based data sharing - Penalties for unauthorized access or use	- Implementing robust cybersecurity measures - Addressing the digital divide in teledentistry access
WHO Guidelines on Digital Health Interventions^68^	Global	- Ethical use of AI in healthcare - Ensuring equity and accessibility in digital health - Promoting interoperability of health systems	- Harmonizing regulations across different countries - Addressing ethical concerns in AI-driven diagnoses

### 4.4 Legal Obligations for Informed Consent and Transparency

Informed consent from patient is not only an ethical, but also a legal imperative of healthcare^69^. The use of AI in patient care adds yet more layers of complexity to informed consent. Legally, patients must be given full disclosure about AI’s role in diagnosis and treatment as well as the risks, benefits and limitations of AI technologies^70^.

Healthcare providers are legally required to deliver content in an accessible way to the patient based on their language skills, culture, and cognitive capacity^69^. Information on informed consent needs to be recorded in a comprehensive manner and in compliance with the law, acknowledging that consent may need to be revisited in case the AI technologies employed or the use of patient data undergo a significant transition. Transparency in AI care is trust building, and it also provides the required legal protection for patient autonomy and rights.

### 4.5 Cross-Border Legal Challenges

The worldwide expansion of teledentistry with AI technology creates major legal challenges because data protection laws differ between nations. Services providers must work through both GDPR and the HIPAA requirements while offering their services across international borders^71^.

GDPR requires all companies handling EU citizens' personal data to follow specific rules on consent, data reduction, and data usage purposes no matter where they process data^71,72^. Under GDPR people receive strong rights including the right to erase personal data and move it between services^71^. HIPAA enforces Protected Health Information (PHI) protection rules for U.S. covered entities and their business partners through all their activities^71^. Despite both regulations requiring protection for sensitive data with security and notification steps, they operate differently across regions and have unique rules^71^. **Table 5**^71-79^summarizes the key differences between GDPR and HIPAA.

**Table 5:** Key Differences Between GDPR and HIPAA This table provides a comparison between the key provisions of the General Data Protection Regulation (GDPR) and the Health Insurance Portability and Accountability Act (HIPAA) that demonstrates their distinctions in terms of scope and enforcement practices. This section offers a brief summary of regulatory effects on data handling for businesses working between the European Union and United States.

Feature	GDPR	HIPAA
Scope of Data	All personal data of EU residents, including health data^71^	Protected Health Information (PHI) in the U.S.^71^
Territorial Scope	Applies to any organization processing EU residents' data, regardless of location^71^	Primarily applies to U.S.-based covered entities and their business associates^71^
Individual Rights	Broader rights, including the right to be forgotten and data portability^71^	More limited rights, primarily focused on access and amendment of health records^71^
Consent	Explicit, opt-in consent required^72,75^	Authorization required for uses/disclosures not related to treatment, payment, or healthcare operations^75^
Data Transfers	Strict rules on transfers outside the EU; requires safeguards like MCCs or adequacy decisions^73,78^	Less restrictive on international data transfers^74^
Data Localization	May be required in certain cases or to simplify compliance^73,77^	Generally not required^77^
Penalties	Up to 4% of global annual turnover or €20 million, whichever is higher^71^	Generally lower, with a maximum of $1.5 million per violation per year^71^
AI Governance	Addresses automated decision-making; EU AI Act sets further requirements for high-risk AI systems^76,79^	No specific provisions for AI, but general privacy and security rules apply

Teledentistry providers serving patients in both EU and US must deal with multiple data compliance rules by creating separate data handling processes for each region^76^. The GDPR regulation about data movement across borders create extra compliance challenges. Data transfers from EU to other countries must follow Model Contractual Clauses (MCCs) or be approved by European Commission adequacy decisions^78^. EU patients need their data to stay inside EU^77^, or require specific consent for cross-border transfers^73^.

GDPR's detailed consent requirements force healthcare providers to revise their HIPAA-based authorization forms to match GDPR's precise standards^80^. Teledentistry providers need to handle additional regulations when they use AI technology because it affects decision-making automation and requires clear algorithm explanations under both GDPR and the upcoming EU AI Act^79^.

Many AI teledentistry companies worldwide adopt multiple strategies to meet the strictest data security requirements from both GDPR and other regulations^80^, using MCCs for data transfers ^78^, and establishing regional compliance partnerships^77^. Several providers choose to store data inside specific regions to make compliance easier^77^.

AI teledentistry faces new challenges because global rules about regulation and privacy keep changing. Countries like India^76^ and Australia are changing their privacy laws, which could make companies face conflicting obligations when managing data. Teledentistry service providers need to adopt flexible and forward-looking compliance strategies. These regulations offer possibilities for harmonization. The World Health Organization's effort for international AI healthcare guidelines^77^ could simplify compliance for stakeholders across multiple jurisdictions.

Compliance with GDPR, HIPAA, and emerging regulations will be crucial for AI teledentistry providers. Effective data governance, global collaboration, and the ethical development of AI have become crucial to ensuring compliance and supporting innovation.

## 5. Economic Considerations in AI-Driven Teledentistry

The implementation of AI technology in teledentistry has significant financial repercussions that includes cost-benefit evaluations, difficulties in adoption, the potential reduction of healthcare costs for marginalized populations, and financial benefits to promote integration. Stakeholders must possess a thorough understanding of these economic factors in order to make educated decisions about investments in AI technologies.

### 5.1 Cost-Benefit Analysis of AI Implementation

A detailed cost-benefit analysis is required to understand the economic viability of AI-enabled teledentistry. While AI can boost efficiency and clinical outcomes, the commercial benefit is uncertain and highly contingent. AI can involve significant upfront training, software and hardware investment. It should also factor ongoing maintenance, upgrades, and technical support^81^.

Increased patient satisfaction, increased patient throughput, and greater diagnostic precision are some potential benefits. But those benefits depend on a number of factors such as the reimbursement models for AI services, or the success of treatments following AI-assisted diagnosis^82^. Economic analyses must include clinical settings, populations and existing healthcare infrastructure in order to calculate return on investment (ROI).

### 5.2 Economic Challenges and Barriers to Adoption

A number of financial barriers could prevent AI from being deployed widely across teledentistry. Initial cost of acquisition and implementation of AI technology is typically expensive, especially for smaller practices with limited budgets^83^. This expense can be compounded even further by the need for substantial investment in employee training and education to leverage AI tools^83^.

Another major barrier is the question of return on investment. Unless there is sufficient evidence of savings or improved patient care, dental practices may not be willing to invest in AI technology^84^. The cost burden is compounded by the added cost of ensuring compliance with law, data security, and other regulatory compliance^83^. Costs of AI-supported services can also be affected by variation in reimbursement strategies because service providers may not receive a fair return on their investments^85^.

### 5.3 Reduce Healthcare Costs for Underserved Populations

AI-assisted teledentistry has the ability to lower healthcare costs while simultaneously improving access to dental care for disadvantaged groups. Using AI technologies allows the delivery of dental care remotely, reducing the necessity for physical infrastructure and facilitating access for medical professionals to patients in remote or resource-constrained areas. Recent cost-minimization research shows that teledentistry, using remote examination instead of conventional visual examination techniques, leads to annual savings of $85 million in wages, travel costs, and equipment expenditures^86^. AI allows early detection and intervention which leads to preventing the progression of dental issues, hence reducing the costs associated with future, more expensive treatments^87^.

Cost savings in clinical workflows can be realized by reducing redundancy while improving efficiency. Artificial intelligence can improve the operational effectiveness of record management, appointment scheduling, and patient screening^88^. However, in order to maximize these advantages, targeted research is necessary to quantify cost savings and develop strategies that address difficulties specific to disadvantaged communities, such as those with limited access to technology and internet connectivity.

### 5.4 Financial Incentives to Promote AI Adoption

Providing financial incentives can encourage the incorporation of AI technologies in teledentistry. The acceptance of AI solutions can be promoted through value-based care models that reward providers for improving patient outcomes and reducing costs. Risk-sharing agreements between dental practices and AI technology providers might mitigate the financial risks and rewards related to the implementation of AI^89^.

Initial investment expenses may be financed through government grants, subsidies, or tax incentives, particularly for practices that cater to underprivileged populations^90^. Financial sustainability can be improved through the implementation of reimbursement models that recognize and employ AI-assisted services^89^. AI-driven teledentistry efforts could be more readily funded and developed through public-private partnerships, which promote innovation while distributing the financial burden.

**Figure 4:**
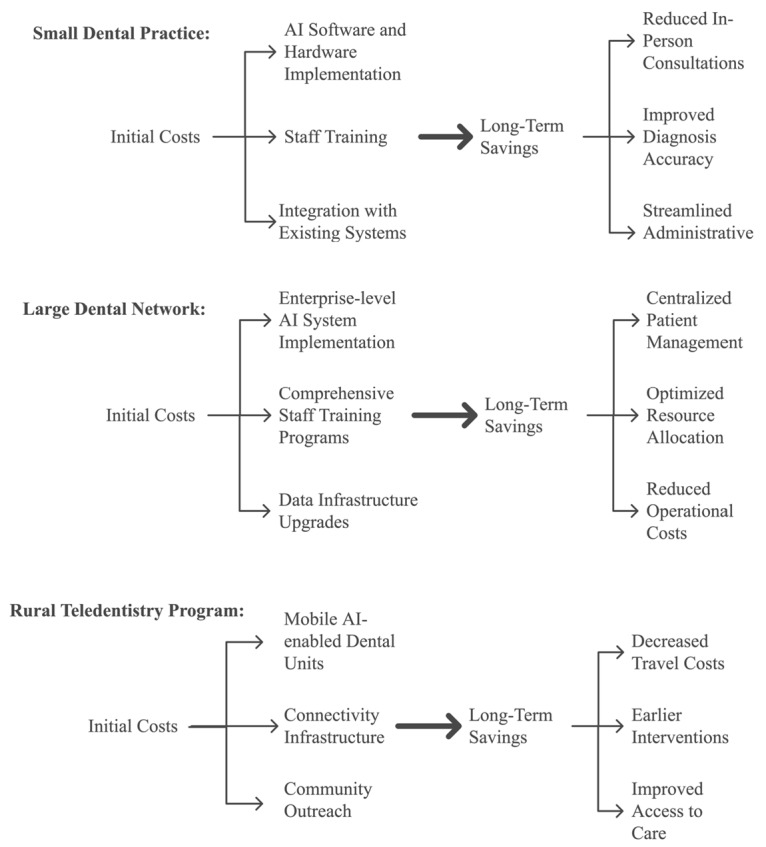
A visual comparison of economic impacts for AI-driven teledentistry Comparison of economic benefits of AI-based teledentistry. This figure compares the economic impact across three contexts: small dental practices, large dental network, and rural teledentistry services. Each setting shows how costs are weighed against returns (increased costs for AI software, staff education, infrastructure upgrades) and benefits (reduced operating costs, better diagnostic precision, increased access to care).

To illustrate the economic considerations discussed, **Figure 4** shows an in-depth comparison of the economic impacts of AI-enabled teledentistry implementation in several different practice environments to help understand the economic impact. It emphasizes the difference in the economics of small dental practices, large dental networks, and rural teledentistry programs. Each case demonstrates how long-term savings and benefits, such as increased diagnostic precision, streamlined operations, and expanded reach to underserved populations, recoup initial investments, from AI system installation and training to infrastructure. The illustration reflects the varied but powerful economic opportunities of AI in teledentistry.

### 5.5 Innovative Financial Models to Increase Accessibility

AI technology adoption in teledentistry faces strong resistance, especially for small clinics with limited budgets, due to high installation expenses including hardware purchases, software licensing, employee training, and system upkeep^91,92^. To address this challenge, several innovative financial models and funding opportunities have been proposed.

One approach would be the use of a pay-per-use model, where clinics are only charged for the AI services that they have used^92^. This system lets clinics use AI services through cloud platforms without buying expensive hardware or software^91^. Similarly, the use of subscription-based pricing helps distribute costs evenly across time while shared savings agreements demonstrate potential benefits in teleradiology when clinics receive a portion of the AI-generated savings. Risk-sharing agreements between teledentistry providers and dental insurers can share risk by teaming up to invest in AI systems for preventive care.

European Union's framework programs offer research and development grants^93^ while tax benefits help reduce clinic costs. Public-private partnerships can fund small clinics by working with technology firms to offer AI solutions at affordable prices through AI-as-a-Service models^94^. Investors in the private sector (e.g., angel investors, seed funding^95^, venture capital) will provide funds for small clinics that show promise through pilot programs. **Table 6**^91-99^ summarizes these innovative financial models and their potential application in teledentistry.

To successfully implement these models, clinics should carry out comprehensive needs assessments ^100^, initiate smaller AI related projects^94^, and create an ecosystem with sufficient infrastructure, regulatory support, and access to funding^94^. Furthermore, it is imperative to define metrics on economic factors (e.g., ROI, cost reduction, increase in revenue), clinical measurements (e.g., patient results, accuracy of the diagnosis), operational parameters (e.g.,

**Table 6:** Innovative Financial Models for AI Adoption in Teledentistry The table details several creative financial models designed to support AI integration in teledentistry with a specific focus on small-scale dental clinics. The table provides details about each model along with its advantages in teledentistry and accompanying relevant examples or references. AI implementation financial barriers are tackled by these models through cost reduction at the start, cost distribution during implementation phases or by sharing financial risks.

Model	Description	Potential Benefits in Teledentistry
Pay-per-use^92^	Clinics pay only for the AI services used.	Reduces upfront costs, aligns expenses with usage.
Subscription-based^92^	Clinics pay a recurring fee for access to AI services, updates, and support.	Spreads costs over time, provides predictable budgeting.
Cloud-based AI Services^91^	AI services are delivered via the cloud, eliminating the need for on-site hardware.	Reduces hardware costs, enhances scalability and accessibility.
AI-as-a-Service (AIaaS)^92^	Access to pre-trained AI models and APIs.	Lowers technical barriers, reduces development time and costs.
Open-source AI Solutions^91^	Utilizing freely available AI tools and platforms.	Eliminates software licensing fees, but may require more in-house expertise.
Shared Savings Agreements^98^	Clinics share a portion of cost savings achieved through AI with the vendor.	Aligns incentives, reduces financial risk for clinics.
Risk-sharing Agreements^97^	Clinics and insurers share the financial risk of implementing AI.	Encourages adoption by distributing financial risk, potential for long-term savings through improved preventive care.
Government Grants^93^	Public funding for AI research, development, and implementation.	Offsets initial investment costs, promotes innovation. Adapted from EU framework programs
Tax Incentives^99^	Tax breaks or credits for clinics investing in AI.	Reduces the financial burden of AI adoption.
Public-Private Partnerships^94^	Collaborations between public entities and technology companies to provide affordable AI solutions.	Leverages private sector expertise and resources, reduces costs for clinics.
Collaborative Approaches^96^	Multiple clinics or providers pooling resources to implement AI.	Distributes costs and expertise, potentially reduces individual clinic investment.
Private Sector Funding^95^	Angel investors, seed funding, venture capital for AI development and scaling.	Provides capital for innovation and growth, particularly for clinics demonstrating value through pilot projects.
Healthcare Innovation Funds^95^	Specialized funds and accelerator programs focused on healthcare technology.	Offers targeted financial and mentorship support for AI implementation in healthcare.

productivity, errors), patient-centered metrics (e.g., patient satisfaction, patient engagement), and staff-related measures (e.g., trainer productivity)^101-105^. Such innovative approaches, when coupled with appropriate impact measurements, make it possible for small clinics to address issues of economic restrictions posed to the adoption of AI in teledentistry and also enhance the quality of care and the performance of the clinic.

## 6. Discussion

AI can enable teledentistry to provide better and more equitable care to patients, make diagnoses more accurate and enable more people to receive dental services, and reach those who are underserved^86,87^. However, our findings demonstrate that important ethical, legal and economic issues will need to be addressed to realize these promises.

### 6.1 Ethical Considerations

Data privacy and security remain key ethical issues as AI in teledentistry handles sensitive patient information across digital platforms^22,53^. Adherence to regulatory frameworks such as GDPR and HIPAA is mandatory^58,106^, but legal compliance alone does not suffice. In practice, AI-driven teledentistry involves data sharing between software vendors, imaging platforms, and cloud services, increasing the number of potential points of exposure. For this reason, privacy-by-design is not only a regulatory necessity but also an ethical imperative.

Informed consent becomes more challenging when AI is involved in screening, diagnosis, or treatment planning. Patients may need to know that AI is being used, what data is being analyzed, how the algorithm is weighting different data points, and to what extent a clinician is supervising the process^19,69^. This question is even more pertinent in teledentistry, where there are fewer opportunities for explanation. The literature argues that transparency is required for patient autonomy to remain intact^18,70^, but the use of black-box models means that patients are, at best, asked to consent without understanding how an AI-driven decision is made. Explainability is therefore an ethical requirement for consent.

Bias in AI systems is often discussed concerning unfair or unequal outcomes^25,56^. From the papers reviewed, we found that bias is specifically linked to dataset issues, such as inadequate demographic representation, disease imbalance, and training data originating from a single institution or patient population. In this context, bias is not merely a vague concern but a direct consequence of dataset creation. Although the use of diverse training data and the monitoring of bias are commonly proposed solutions^26,27^, these efforts will only be effective if demographic and clinical variability is captured during development and external validation.

The increasing deployment of AI in clinical decision-making also provokes important questions about accountability, transparency, and professionalism^107,108^. While most papers concur that AI should not replace clinical expertise^40,41,109^, this creates a critical dilemma. The diagnostic burden motivates a desire for automation. Yet, when models are described as highly accurate or time constraints discourage clinical review, there is a risk of unquestioning reliance. Human-in-the-loop techniques (confidence thresholds, warning flags, clinician verification) are often advocated as solutions. However, if clinicians routinely accept the output without scrutiny, this human-in-the-loop approach risks becoming a human-along-for-the-ride. Over time such scenarios may contribute to deskilling. Ensuring an ethical deployment of AI requires not only human oversight, but human oversight that is not a rubber-stamp.

### 6.2 Legal Considerations

The legal status of AI in teledentistry is in flux and jurisdiction-dependent but all AI-based treatment requires lawful processing of personal data under data protection regulations such as GDPR^58,60^. Algorithmic explainability and transparency will be crucial in adjudicating legal issues such as artificial intelligence-driven diagnosis or treatment planning^108^.

Organizational liability can be an issue in the case of AI systems that misdiagnose or recommend incorrect treatments^58,59^, and these possibilities necessitate clearly defined notions of accountability among the AI developers, dental professionals and healthcare organizations.

### 6.3 Economic Considerations

Although it could reduce the costs and improve access to dental care in underserved populations^86,88^, use of AI is likely to be economically feasible only in a complex and context-dependent way^81^. High capital expenditure (costs associated with purchasing technology, infrastructure and training) is a barrier for dentists with smaller practices^83^; conversely, return on investment and pricing models are unclear, given

that AI won’t receive reimbursement from insurance companies until it can provide reliable diagnoses^84,85^.

Financial drivers such as grants, subsidies and more favorable reimbursement models could spur integration^89,90^, while public-private partnerships or tax incentives could lighten financial burdens^110,111^.

### 6.4 Risk Assessment, Mitigation, and Cybersecurity in AI-Driven Teledentistry

The use of AI technology in teledentistry will help both patients and healthcare providers achieve better patient care and improve operational efficiency. The integration of AI into teledentistry creates new safety concerns that need proper monitoring and control. **Table 7**^112-119^ summarizes major risk areas and recommended mitigation strategies.

**Table 7:** Risk Assessment and Mitigation Strategies for AI in Teledentistry The table details primary risks of AI integration into teledentistry systems with mitigation strategies to address them and supporting references. The document reveals several potential challenges in AI healthcare systems concerning diagnostic accuracy, the protection of data, the fairness of algorithms and the role of human expertise.

Risk / Threat	Description	Key Mitigation Strategies
Diagnostic Errors^113^	AI systems, though often accurate, can produce false positives or negatives, potentially resulting in delayed or incorrect treatments.	- Adopt human-in-the-loop workflows, where a qualified dentist reviews AI-generated diagnoses. - Retrain models regularly on new, diverse data to reduce drift. - Establish clear protocols for low-confidence outputs or disagreements between the AI and dentist.
Data Breaches^113,116^	Handling large volumes of sensitive patient data makes AI-based teledentistry systems attractive targets for cyberattacks, risking patient privacy and possible financial or reputational harm.	- Use robust encryption for data in transit and at rest. - Implement multi-factor authentication for system access. - Conduct regular security audits and penetration testing to detect and address vulnerabilities. - Ensure regulatory compliance (e.g., HIPAA, GDPR).
Algorithmic Bias^112^	Biased training datasets can perpetuate or amplify healthcare disparities, leading to unfair or inaccurate outcomes for certain demographic groups.	- Utilize diverse, representative datasets for training. - Audit AI performance across subgroups (age, gender, ethnicity, etc.). - Employ fairness-aware ML techniques that explicitly account for bias during development.
Overreliance on AI^114^	Relying too heavily on AI can diminish clinicians’ critical judgment and lead to a “deskilling” effect in dental professionals.	- Emphasize that AI supplements clinical expertise, rather than replacing it. - Provide ongoing training on AI capabilities and limitations. - Maintain manual diagnostic protocols alongside AI-supported workflows.
Regulatory Compliance^115^	Evolving AI regulations can challenge teledentistry providers to meet new standards across different jurisdictions and sectors.	- Stay informed about local, national, and international regulatory changes. - Engage with regulatory bodies and industry groups to shape AI standards. - Develop flexible governance frameworks that adapt to new regulations.
Integration Challenges^114,115^	Incorporating AI tools into established dental workflows may disrupt procedures and require updates to existing systems.	- Perform compatibility testing before full implementation. - Provide comprehensive staff training on AI-enhanced workflows. - Establish clear technical support protocols for troubleshooting and maintenance.
Adversarial Attacks^117^	Malicious alterations to input data (e.g., radiographs) can deceive AI models, leading to misdiagnoses or manipulated outcomes	- Adopt adversarial training (exposing AI to tampered inputs) to build robustness. - Regularly update AI models with diverse data sets. - Maintain human oversight to detect anomalies AI might miss.
Malware and Ransomware^118^	Attacks can disrupt teledentistry services, compromise patient data, and cause operational downtime or financial loss.	- Employ anti-malware/anti-ransomware solutions. - Conduct security awareness training for staff to prevent phishing and social engineering. - Implement data backup and recovery systems for swift restoration after an attack.
HIPAA Security Rule Compliance (U.S. only)^12,119^	Teledentistry providers in the U.S. must adhere to HIPAA’s administrative, physical, and technical safeguards for protecting electronic protected health information (ePHI).	- Administrative: Conduct ongoing risk assessments, security awareness training, and contingency planning. - Physical: Control access to systems and data, enforce workstation security. - Technical: Enable access controls, audit logs, integrity checks, and transmission security.

Ongoing Monitoring and Adaptation: As AI in healthcare evolves rapidly, teledentistry providers should regularly monitor and adjust their security practices to protect patient data. Key strategies include:

· Continuous Monitoring: Regularly track AI performance and security logs while adding new threat intelligence updates.

· Staff Training: Schedule regular training sessions to share new security threats and industry standards with team members^118^.

· Staying Informed: Keep updated on new security vulnerabilities, regulatory changes and technology changes that may require adjustments in protocols.

· Adaptive Protocols: Adjust security measures and AI architecture following new safety standards plus information gained from real-world incidents.

· Industry Collaboration: Join professional networks to share and receive threat intelligence and mitigation strategies with peers.

The adoption of broad plans for risk assessment, mitigation, and cybersecurity allows teledentistry providers to enhance the safety, reliability, and public trust of their AI-driven solutions, thus encouraging responsible and ethical development in teledentistry.

### 6.5 Real-World Examples of AI Implementation in Dentistry

The use of AI in dentistry, including teledentistry, is still in its infancy, yet current examples demonstrate that its impact goes beyond technical automation. They influence clinical decision-making, continuity of care, patient engagement, and access to services, especially in contexts where in-person care is challenging. Instead of being mere tools, they have the potential to alter the constancy with which clinicians recognize disease, the timeliness of patient intervention, and the diligence of their follow-up.

### 6.5.1 AI-Powered Diagnostic Tools

Several AI tools have been developed to assist in dental disease detection and to reduce variability in image interpretation. Their value could extend beyond mere efficiency, potentially impacting clinical outcomes by facilitating earlier and more consistent detection.

· VideaHealth’s AI algorithm, an FDA-cleared software for dental caries detection, has been reported to reduce missed caries diagnoses by over 40% and false caries diagnoses by almost 15%^120^. Clinically, this could reduce diagnostic variability between practitioners and enable earlier intervention, potentially shifting care away from delayed restorative treatment.

· Overjet’s Dental Assist software uses deep learning to detect periodontal disease and bone loss in dental radiographs^120^. By standardizing the detection of periodontal changes, it has the potential to save lives by improving treatment planning and follow-up in cases where progression might be overlooked.

· Pearl’s Second Opinion uses computer vision to detect multiple dental findings: caries, calculus, inflammation, crowns, fillings, root canals, bridges, and implants ^120^. These applications may enhance patient care by acting as a second set of eyes to reduce missed findings, although their value still relies on clinician interpretation.

Together, these tools suggest that AI-assisted diagnostics could standardize care by reducing interpretive variability, enhancing detection, and facilitating earlier intervention—provided they generalize effectively.

### 6.5.2 Remote Monitoring and Teleorthodontic

Remote monitoring systems illustrate how AI may alter patient care not only by detecting disease, but by extending clinical oversight between in-person visits.

Dental Monitoring (DM)is a platform that combines AI with remote dental care to enable partial automation of orthodontic treatment monitoring^121^. By submitting images and receiving feedback between office visits, patients experience a more continuous form of supervision than is possible with conventional appointments.

### 6.5.3 AI-Enhanced Patient Communication and Screening

Some AI applications are designed to improve initial access, communication, and early screening rather than direct diagnosis alone.

· AI-powered chatbots: These tools enable patients to describe symptoms, upload pictures, and receive preliminary advice or educational content without an office visit^122^. Their primary impact is in enhancing patient access and communication, potentially prompting them to seek care sooner, though they cannot substitute for a clinical evaluation.

· MeMoSa mobile screening app: This app achieved 80% accuracy in detecting oral lesions from images^122^. Its significance lies in its potential use in remote or underserved areas, where early screening could guide patients towards timely evaluation and help prevent delayed referrals.

These applications suggest that AI may support patient care not only through diagnosis, but also through earlier entry into the care pathway, especially in communities with limited dental access.

### 6.5.4 Pilot Programs and Public Health Initiatives

Public health initiatives provide stronger evidence of patient-care relevance because they report effects on actual service delivery and oral health outcomes.

· **Iowa’s Virtual Dental Home Pilot Project**: This asynchronous teledentistry initiative in two Iowa long-term care facilities enabled residents to receive preventive and disease control services on site. The program led to a reduction in untreated caries and an increase in arrested caries^123^. These findings suggest that AI-supported or teledentistry-enabled care models may improve outcomes in vulnerable populations by shifting care toward earlier prevention and disease stabilization.

· Dental Health Services Victoria (DHSV): During the COVID-19 pandemic, DHSV implemented a patient-initiated teledentistry model in Victoria, Australia. Between May 2020 and April 2021, 2,492 patients used telehealth services, and 87% reported that the care met their needs^124^. This indicates that remote care models can maintain patient access and perceived adequacy of care during periods when traditional delivery is disrupted.

These programs show that the relevance of AI and teledentistry extends beyond technical feasibility and includes measurable effects on access, continuity, and patient-centered outcomes.

### 6.5.5 Integration into Clinical Workflows

AI is increasingly being incorporated into routine dental workflows, and its clinical value depends on whether these integrations improve decision-making without weakening clinician oversight.

· Diagnostic Imaging Analysis: AI has been used to detect periapical lesions and assess endodontic outcomes with notably high reported accuracy^125^. Their value lies in assisting with image review and focusing attention on potentially missed findings.

· Treatment planning: AI-based 3D imaging and virtual planning tools can support implant placement and prosthetic-driven planning, which may improve procedural accuracy and workflow efficiency^126^.

· Clinical documentation: Natural language processing tools can help generate reports and documentation^127,128^. Although such tools might free up clinicians’ time and allow them to focus more on patient care, they still require thorough review to verify their completeness and clinical accuracy.

Taken as a whole, these real-world examples indicate that AI has the potential to impact patient care by: improving diagnostic consistency, extending monitoring beyond visits, enhancing triage and access, and supporting workflow efficiency - but only when implemented with clinician oversight and validated in real practice settings.

### 6.6 Strengths and Limitations

This scoping review offers a comprehensive synthesis of the ethical, legal, and economic dimensions of AI in teledentistry, yet several limitations must be acknowledged to contextualize the findings.

· Language and Publication Bias: The restriction of the search to English-language publications, while encompassing a large proportion of global research, likely excludes relevant regional developments and legal frameworks published in other languages, particularly in rapidly advancing non-English speaking dental markets.

· Inclusion of Grey Literature: Although including grey literature was crucial to capture the most up-to-date regulatory and policy documents from government and international bodies, these sources often lack the rigorous peer-review process of academic journals. This may impact the quality of evidence synthesized about implementation.

· Rapid evolution of the AI field: The field of artificial intelligence is evolving at an unprecedented pace, with new algorithms, FDA clearances, and regulatory acts — such as the EU AI Act — emerging even as this review was being finalized. As such, specific AI performance metrics or legal compliance may represent a “snapshot in time” that requires frequent updating to keep pace with technological shifts.

· Methodological Limitations: This review employed two independent reviewers and a third-party conflict resolution to minimize bias; however, the thematic grouping is at the discretion of the authors regarding what constitutes ‘responsible innovation’. In addition, the heterogeneity and lack of standardization in economic reporting within the dental literature precluded a quantitative meta-analysis of the cost savings associated with AI.

### 6.7 Comparison with Other Work

This review’s findings align with prior literature emphasizing data security, informed consent, and bias as key issues for AI in healthcare^22,25^, yet it also highlights how these concerns overlap in the context of teledentistry, where remote care, digital imaging, and platform communication introduce additional challenges. Rather than being disparate issues, privacy, consent, bias, and cost are interconnected and may collectively determine whether AI can be deployed responsibly.

One important insight from this review is that bias is not only an abstract issue of fairness to be monitored, although that is important, but also a consequence of identifiable failures in training and test data. Bias has been flagged as an ethical concern in several previous reviews^25,56^, but the evidence here suggests that performance disparities are frequently driven by limited demographic coverage, single-site datasets, and a lack of external validation. This is particularly relevant for teledentistry, where AI may be deployed across populations with differing disease profiles, image quality, and access conditions.

The review also underscores a continuing dilemma between efficiency and professional oversight. Many papers conclude that maintaining a human-in-the-loop is necessary for ensuring accountability^40,41,109^. However, this review finds that this approach addresses only half the problem. Without robust clinical governance, relying on confidence thresholds and clinician review to prevent blind automation also risks fostering overreliance. In this context, the literature identifies a critical, unresolved issue: AI may streamline workflows but at the cost of eroding professional expertise if the safeguards are merely performative.

Economic considerations reveal a similar disconnect between discourse and reality. High initial costs, ROI uncertainty, and the need for cost-benefit analysis have been cited as barriers in previous work^81,83^ – a finding supported by this review. However, the solutions proposed in the literature diverge more sharply. Subscription, pay-per-use, and AI-as-a-service models are frequently suggested as means to alleviate adoption costs, particularly in smaller and underserved practices. Yet, if clinics must still incur costs for digital infrastructure, imaging equipment, training, integration, and regulatory compliance, then the adoption barrier remains. Payment flexibility may improve affordability – but only in contexts where the technical capacity already exists.

Compared to previous studies that have focused on either AI performance or ethical considerations in health care, this review highlights the importance of considering all these elements in combination. The literature indicates that adopting AI in teledentistry will only be responsible if the datasets are representative, oversight preserves professional autonomy, and the adoption scenarios can be realized in practice.

### 6.8 Gaps and Opportunities

Further research particularly needs to develop internationally standardized legal frameworks applicable to AI in teledentistry^60,61^. Deep economic evaluations are needed to quantify cost savings and efficiencies, especially for vulnerable communities^82,87^. It will remain challenging to address these shortcomings, but doing so will facilitate safe, responsible and equitable implementation of AI technologies into dental practice.

## 7. Future Research Directions and Policy Recommendations

The rapid growth of AI in teledentistry requires both ongoing research and better policies to properly use this technology in dental care. Key priorities include continuous monitoring and improvement, regular ethical audits, multi-stakeholder collaboration, efficient software updates, and clear policy guidelines.

### 7.1 Continuous Monitoring and Improvement of AI Systems

Future research should be focused on establishing procedures for continuous evaluation of ongoing system performance and retraining using new patient data, alongside detecting data drift to identify the shifts in input distributions^129^. Real-time dashboards should be used for tracking performance metrics such as accuracy, sensitivity, and specificity across diverse patient subgroups to identify performance gaps^130^. Moreover, error logs and feedback systems (which include clinician feedback) offer further opportunities for improvement^130^. Automated alerts in the case of performance shortfalls^129^ and uncertainty assessment to identify intricate cases enhance reliability protection. Benchmarking performance against gold-standard diagnoses and cross-validation in various populations contribute to greater model generalizability^28,44^.

### 7.2 Regular Audits and Ethical Considerations

The optimal frequency and scope of systematic reviews or audits remains an open discussion, comprehensive reviews conducted on a quarterly basis coupled with rapid checks on biannual basis should offer a practical framework. These assessments have to be undertaken in the domains of ethical and legal compliance, data quality and security, and possible biases in the model performance evaluation. Multidisciplinary review boards comprising AI professionals, dentists, ethicists, lawyers, and patient advocates can ensure adequate balance in governance.

### 7.3 Multi-Stakeholder Collaboration and Knowledge Sharing

The creation of effective multi-stakeholder forums is important for sharing best practices, findings from new studies, as well as other updated information^24^. Interdisciplinary working groups, modeled after successful initiatives like the European Huntington's Disease Network^131^, can be formed to address specific issues in AI powered teledentistry. Forums for open science leadership, like those organized by the Bill and Melinda Gates Foundation^132^, can encourage sharing of knowledge among different stakeholders, including participants from both developed and developing countries.

Dental associations or regulatory bodies can form AI governance committees to monitor the unethical use of AI, guidelines development, and checking for discrimination. A collaborative research consortium can offer support and combined expertise for their large-scale validation studies, whilst public private partnerships, like those suggested in the Human Exposome Project^133^, can foster innovation and development in a responsible manner. Active patient advocacy groups should also be on the forefront to ensure that AI solutions offered are relevant to what patients really want and need.

### 7.4 Efficient Software Updates and Implementation

Future research should address strategies to update software without causing interruptions to patient care. This may include robust version control and patch management^134^, scheduled maintenance during off-peak hours, and redundant systems with failover mechanisms. The implementation of automated testing through CI/CD pipelines helps teams find and fix problems quickly^134^. Additionally, clear rollback procedures with backups of previous stable versions should be in place^135,136^. A formal change control system needs to combine a team of experts who review risks plus effective communication methods

### 7.5 Policy Recommendations

Based on this review, we propose the following policy recommendations for the responsible adoption of AI in teledentistry, addressed to regulators, health systems, and clinicians alike.

· Require periodic assessment, focusing on diagnostic accuracy, bias, and ethical and legal compliance. Performance should be monitored, including external validation across populations and care settings.

· Define clear data-governance policies related to data quality, privacy, security, storage, and sharing in AI-driven teledentistry, ensuring compliance and patient trust.

· Promote interoperability standards to support integration of AI tools with imaging, EHRs, and telehealth. Poor interoperability can limit clinical utility even if the algorithm performs well.

· Support affordable adoption models for smaller clinics and underserved communities. While subscription, AI-as-a-service, or pay-per-use models may lower some cost barriers, policy support may still be required.

· Develop clinical guidance mandating AI provide confidence scores or uncertainty information where possible. This would allow clinicians to recognize when further review is necessary and avoid over-reliance on automated results.

· Strengthen patient-centered transparency in consent. Consent should not be a mere legal safeguard. Wherever practicable, clinicians should describe the role of AI in the diagnosis or monitoring in text that patients can understand, including the use of explainability tools where appropriate.

· Address clinician overreliance and deskilling. Implementation policies must include training requirements that emphasize AI as an aid, not a replacement, for clinical judgment. Embed AI literacy in dental curricula and continuing professional development so clinicians understand how to critically evaluate AI outputs rather than blindly trust them.

· Foster multi-stakeholder collaboration to share evidence, implementation experience, and evolving standards for responsible use.

· Prioritize funding for teledentistry AI research that addresses equity, external validation, patient outcomes, and access to care over technical performance.

· Encourage the education of dental professionals on the ethical, legal, and clinically effective use of AI in practice.

In summary, policy development must address not only systems regulation but also the practical conditions for safe usage in day-to-day clinical care.

## 8. Conclusion

This review, unlike previous studies, addresses the ethical, legal, and economic considerations of AI in teledentistry rather than its technical aspects. It highlights transparency, consent, regulation, and cost as central themes and summarizes the factors that will influence its adoption.

AI-driven teledentistry provides an opportunity to enhance the efficiency, accessibility, and personalization of dental care. It has the potential to improve diagnostic accuracy, optimize workflows, and bridge gaps in access using technologies like machine learning, generative adversarial networks (GANs), and the Internet of Things (IoT). However, it also presents several challenges.

Ethically, algorithmic bias, privacy, and informed consent demand policies safeguarding patient autonomy and fairness. Legally, data protection regulations and liability frameworks must be addressed. Economically, AI may save costs through earlier detection and increased efficiency but requires significant initial investment.

To tackle these issues, dentists, technologists, ethicists, policymakers, and economists must collaborate. Financial support, through grants, subsidies, and reimbursement schemes, could facilitate adoption in underserved contexts.

Overall, AI-assisted teledentistry has the potential to enhance dental care delivery and outcomes. Its responsible deployment requires a holistic approach that addresses ethical, legal, and economic considerations.
